# Myocarditis in an FIP-Diseased Cat with FCoV M1058L Mutation: Clinical and Pathological Changes

**DOI:** 10.3390/ani14111673

**Published:** 2024-06-03

**Authors:** Chiara Guarnieri, Luca Bertola, Luca Ferrari, Cecilia Quintavalla, Attilio Corradi, Rosanna Di Lecce

**Affiliations:** 1 Department of Veterinary Science, University of Parma, 43126 Parma, Italy; chiara.guarnieri1@unipr.it (C.G.); cecilia.quintavalla@unipr.it (C.Q.); rosanna.dilecce@unipr.it (R.D.L.); 2 Department of Veterinary Medicine, University of Milan, 26900 Lodi, Italy; luca.bertola@unimi.it; 3 Mouse and Animal Pathology Laboratory (MAPLab), Fondazione Unimi, 20139 Milano, Italy

**Keywords:** cat, myocarditis, FIP, FCoV *S* gene mutation, M1058L biotype

## Abstract

**Simple Summary:**

Feline infectious peritonitis (FIP) is a very common coronavirus (FCoV) infectious disease in the feline population. FIP infection is a cause of death in cats and is widespread in domestic cats. FIP can have an acute or chronic clinical form, also known as effusive or non-effusive FIP, respectively. The typical lesion found in the acute form is an effusion in the thoracic and/or abdominal cavities, while collection in the pericardial sac is rare. The chronic form is characterized by pyogranulomatous necrotizing lesions in organs such as the kidney, liver, intestine, lung, eyes, skin, and central nervous system. FIP does not represent a zoonotic risk. The reported case is an uncommon pyogranuloma found in the myocardium in a cat with clinical heart dysfunction. The most important histopathological finding was the myocarditis/myocardial necrosis associated with the presence of *S* gene-mutated FCoV (M1058L biotype). This is the first described case of myocarditis in an FCoV/FIP M1058L biotype-positive cat.

**Abstract:**

An 8-month-old intact male domestic shorthair cat was referred to the Emergency Service of the Veterinary Teaching Hospital (VTH) of the Department of Veterinary Science of the University of Parma (Italy) from the Parma municipal multi-cat shelter, during the winter season (January 2023), for lethargy, anorexia, hypothermia, and hypoglycemia. At the VTH, upon cardiologic examination, an increase in heart rate, under normal blood pressure conditions, was detected. Signalment, clinical history, basal metabolic panel (BMP), ultrasound investigations, and cytological findings were all consistent with a diagnosis of feline infectious peritonitis (FIP). FIP was confirmed in the effusive abdominal fluid by a molecular genetic test (real-time PCR for feline coronavirus RNA). The molecular genetic investigation also detected an FCoV *S* gene single-nucleotide mutation: biotype M1058L. At necropsy, an effusive collection was recorded in the abdomen, thoracic cavity, and pericardium sac. White parenchymal nodules, of about 1 mm diameter, were found on the surface and deep in the lungs, liver, kidneys, and heart. Histopathology revealed the typical FIP pyogranulomatous vasculitis and IHC confirmed the presence of the FIP virus (FIPV) antigen. The most relevant histopathological finding was the myocarditis/myocardial necrosis associated with the presence of the *S* gene-mutated FCoV (M1058L biotype). This is the first case of myocarditis in a cat positive for the FCoV/FIP M1058L biotype. Further studies are necessary to support the mutated FCoV M1058L biotype, as an uncommon, but possible, causative pathogen of myocarditis in FCoV/FIP-positive cats. Studies including several FCoV/FIP M1058L-positive cases could allow us to make a correlation with heart gross pathology, histopathology, and immunolocalization of the FCoV/FIP M1058L biotype in the myocardium. The investigation will potentially allow us to determine the effective tropism of the FCoV/FIP M1058L biotype for myocardiocytes or whether myocardiocyte lesions are evident in the presence of concomitant causes related to the patient, its poor condition, or external environmental distress such as cold season, and whether the aforementioned concomitant events are correlated.

## 1. Introduction

Feline coronavirus (FCoV) is a frequent, often asymptomatic, pathogen responsible for intestinal infections in cats, found worldwide in domestic and wild felids [1]. FCoV comprises two genotypes, FCoV type I (FCoV-I) and FCoV type II (FCoV-II), and both FCoV genotypes have two biotypes: feline enteric coronavirus (FECV) and feline infectious peritonitis coronavirus (FIPV) [2,3]. The two biotypes show a well-defined clinical course and specific pathological changes. FECV infects the intestine, usually with an asymptomatic course or associated with the presence of mild diarrhea.

FECV replicates in epithelial cells of the middle–lower small intestine as well as in the caecum, then spreading through the blood or lymphatic vascular system to the lymphoid tissue (mesenteric lymph nodes and palatine tonsils) and to the upper respiratory tract [4,5,6]. Inside macrophages, FCoV can mutate into FIPV [5,7,8,9], and afterwards, the systemic infection is transmitted by infected monocytes [5,6]. FIPV infection, the most aggressive and lethal form of FCoV infection, is well known as feline infectious peritonitis (FIP) and manifests in its two classic clinical–pathological forms: wet (effusive) or dry (non-effusive) [3,10].

Historically, FIP was the first recognized form of FCoV infection in cats, described for the first time in 1966 as infective peritonitis in cats [11]. FECV infection in cats was recognized and described fifteen years later, in 1981 [5,12]. Kittens and young cats aged less than one year are more susceptible to FCoV infection and develop FIP as fatal disease [2].

The cardiac localization of FCoV/FIP was reported in one case by Ernandes and coll [13]. It is important to highlight that Yoshida and coll [14] reported a dilated cardiomyopathy-like disease related to FIP virus with myocardial scar lesions, without viral genotyping.

FIP viral mutation is related to the *S* gene and differs in two amino acids in the protein chain, at 1058 methionine (M) to leucine (L) or at 1060 serine (S) to alanine (A). The *S* gene mutations at 1058 and 1060 are related to the cellular tropism [5,15,16,17,18]. Positivity for the FIPV M1058L variant in the heart was detected by RT-qPCR in the absence of inflammatory lesions [19].

The present study investigated gross pathology and histology myocardial lesions in an FIP-diseased cat that was infected by the FCoV biotype with the *S* gene mutation M1058L, considering that the previous cases reported by Yoshida and coll [14] and Ernandes and coll [13] did not test the presence of any FCoV variant.

## 2. Detailed Case Description

An 8-month-old intact male domestic shorthair cat, weighing 2 kg, was referred to the Emergency Service of the Veterinary Teaching Hospital (VTH) of the University of Parma (Italy) from the Parma municipal multi-cat shelter on 25 January 2023, for lethargy, anorexia, hypothermia, and hypoglycemia. The cat was found in the territory when it was 2 months old and then underwent regular vaccination and was dewormed with a fipronil/s-methoprene/eprinomectin/praziquantel spot-on solution for small cats and kittens (Broadline™, Boehringer Ingelheim, Ingelheim am Rhein, Germany). Since then, the cat had always lived with other rescued cats. There was nothing to report in the medical history except for occasional diarrhea.

### 2.1. Clinical Exam at the VTH

Physical examination showed icterus of mucous membranes and hypothermia (36 °C). The heart rate was 220 bpm and the systolic blood pressure, measured by using a SunTech Vet30 Veterinary Monitor (SunTech, Morrisville, NC, USA), was 120 mmHg.

### 2.2. Blood Analysis

A complete blood count (CBC) and basic metabolic panel (BMP) were performed at the VTH clinical pathology laboratory (Table 1 and Table 2).

Severe hypoglycemia was recorded. The values of the enzymes CK and LDH were significantly out of range, higher than 15 times the upper range limit (Table 2). 

Other out-of-range BMP data were consistent with hepatocellular damage: AST, ALT and bilirubin, with an albumin/globulin ratio of 0.3 (Table 2). CBC data indicated neutrophilic leukocytosis (Table 1).

### 2.3. Ultrasound Investigation and Effusion Analysis

On ultrasound examination, abdominal effusion, diffuse peritonitis, echo signs consistent with hepatopathy, splenopathy, lymphadenopathy of mesenteric lymph nodes, and nephropathy were found. 

The abdominal effusion was sampled and analyzed for the physical aspect and protein content and smeared for cytological examination (Table 3). The abdominal effusion was clear, viscous/sticky and straw-yellow, with a total protein concentration of 5.7 g/dL (globulins 4.3 g/dL) and an albumin/globulin ratio of 0.3. 

Cytology revealed the presence of non-degenerated neutrophils with vacuolated hyperbasophilic cytoplasm, macrophages, reactive mesothelial cells, and scattered erythrocytes embedded in a granular proteinaceous eosinophilic background.

A specimen of abdominal effusion was stored in a tube, without anticoagulant, for FCoV molecular genetic testing (real-time PCR) and an *S* gene genetic mutation test at IDEXX laboratories (Table 4).

The abdominal fluid was RT-PCR-positive for FCoV RNA, positive for M1058L gene *S* mutation, and negative for S1060A gene *S* mutation.

### 2.4. Therapy

A bolus of glucose solution and a bolus of crystalloids at 5 mL/kg were administered; then, fluid therapy of 2 mL/kg/h was set to counteract severe hypoglycemia and restore and maintain the normal hydration status.

### 2.5. Euthanasia, Post-Mortem Examination, and Histopathological and Immunohistochemical Investigations

Owing to the worsening of the clinical signs and unfavorable long-term prognosis, the cat was humanely euthanized in accordance with American Veterinary Medical Association (AVMA) Guidelines 2020 [20], and a post-mortem examination was performed. At necropsy, the heart was weighed (21.1 g) and many tissue specimens were collected from organs/apparatuses and fixed in 10% *v*/*v* buffered formalin. Formalin-fixed paraffin-embedded (FFPE) 4 μm sections were prepared on polylysine-coated slides for routine histological staining (hematoxylin–eosin; H&E) and immunohistochemistry (IHC). IHC was performed on FFPE sections of the myocardium, kidneys, lungs, lymph nodes, and liver.

IHC FFPE 4 µm sections underwent deparaffinization and heat-induced epitope retrieval (HIER) in a water bath for 30 min at 100 °C, pH 9.0 (Dewax and HIER Buffer H, Lab Vision™, cat. TA-999-DHBH, Thermo Fisher Scientific, Waltham, MA, USA). The slides were rinsed in PBS and placed into an autostainer (Lab Vision™ Autostainer 480S-2D, Thermo Fisher Scientific) after application of PAP Pen Liquid Blocker (Daido Sangyo Co., Ltd., Tokyo, Japan). Endogenous peroxidase activity was blocked by incubating sections with 3% hydrogen peroxide (H_2_O_2_) for 10 min. The slides were rinsed, incubated with PBS containing 10% normal horse serum (NHS) for 30 min at room temperature (RT) to prevent unspecific background staining, and then incubated for 1 h and 30 min at RT with a primary antibody (FIPV, feline coronavirus, clone FIPV3-70, code MCA2149, BioRad, Hercules, CA, USA). Sections were subsequently rinsed with PBS and incubated with a biotinylated secondary antibody (horse anti-mouse, cat. BA-2000, Vector Labs, Burlingame, CA, USA) and labeled using an avidin–biotin–peroxidase procedure (VECTASTAIN^®^ Elite ABC-Peroxidase Kit Standard, cat. PK-6100, Vector Labs). The immunoreaction was visualized with a 3.3′-diaminobenzidine substrate (DAB, Peroxidase DAB Substrate Kit, cat. SK-4105, Vector Labs) (Table 5). Sections were counterstained with Mayer’s hematoxylin, dehydrated in a graded alcohol series, and cover-slipped with a resinous mounting medium. Adequate positive (renal pyogranulomatous inflammation of an FIP-positive case) and negative controls (omission of primary antibody) were included in each immunolabelling assay.

**Figure 1:**
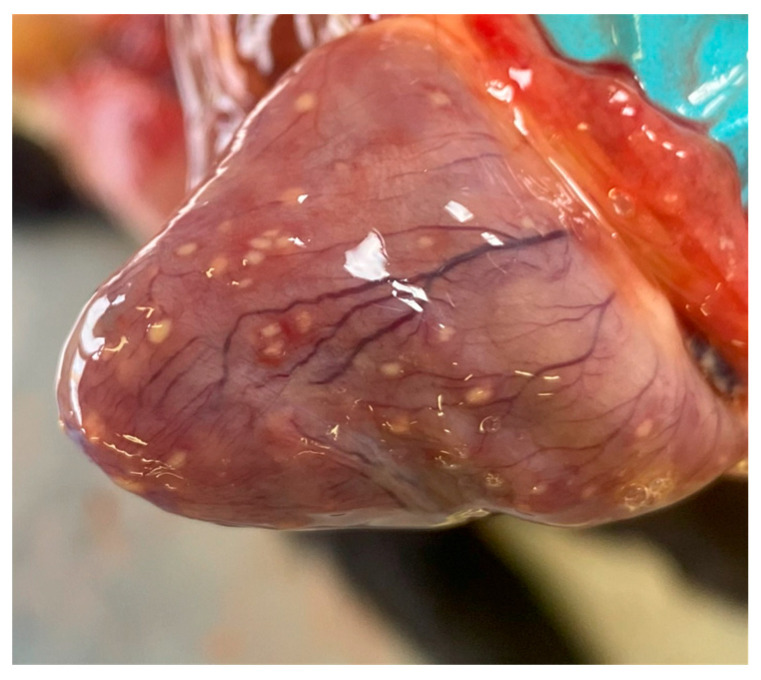
Heart. Multiple well-circumscribed white nodules were observed on the epicardial surface.

During the post-mortem examination, an abundant viscous sero-fibrinous effusion was recorded in the abdominal, thoracic, and pericardial splanchnic cavities. The effusion appeared yellowish and flocculent for the presence of a sediment of fibrin. Multiple well-circumscribed, firm, and white nodules of about 1 mm in diameter were found on the surface and deep in the parenchyma of the lungs, liver, and kidneys. The same nodules were observed on the epicardium and myocardium (Figure 1), and in the intestinal peritoneum as well as in the intestine wall. Multiple mesenteric lymph nodes were enlarged and firm.

**Figure 2:**
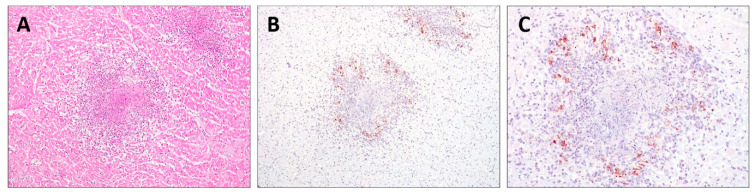
Histopathology and immunohistochemistry (IHC) of the heart. (**A**) Pyogranulomatous inflammation with a central core of necrosis (H&E, 200×); (**B**,**C**) FIPV-immunopositive macrophages were observed at the periphery of foci of myocardial necrosis (IHC, 200× and 400×, respectively).

Histopathology revealed pyogranulomatous infiltration involving several organs: kidneys, lungs, myocardium, lymph nodes, liver, and intestine. Multifocal to coalescing areas of pyogranulomatous vasculitis composed of macrophages, neutrophils, and rarer lymphocytes were found, often centered on central cores of lytic necrosis (Figure 2A). The findings were compatible with a diagnosis of FIP. The IHC of the FFPE sections of the myocardium, kidneys, lungs, lymph nodes, and liver immunostained FCoV-positive cells was morphologically consistent with macrophages at the periphery of necrotic foci (Figure 2B,C).

## 3. Discussion

FCoV infection is usually asymptomatic or causes mild enteritis that is unresponsive to supportive treatment [21,22]. If feline macrophages fail to eliminate the virus, it replicates within their cytoplasm and FIP develops [21,22]. A stressful environment such as that of multi-cat households or shelters and the sharing of a litter box with FCoV-infected cats increase the likelihood of FCoV mutation to FIPV [23]. FIP is an immune-mediated disease and is a common infectious cause of death in cats [24,25]. The percentage of FCoV-infected cats that develop FIP is estimated to be 5–12% [26,27]. When FIP develops, FCoV infection induces pyogranulomatous vasculitis and/or serositis [28], leading to a disease that may or may not be accompanied by high-protein effusions. FIP associated with effusion development, especially in the abdominal cavity, is the most common type of FIP [29]. A range of other systemic signs can occur, such as pyrexia, inappetence, lethargy, abdominal lymphadenopathy, ocular signs, and/or neurological signs [30,31,32,33,34,35,36]. 

The cat of the present study was referred to the VTH, during the winter season, under hypothermia conditions. The detected hypothermia is classifiable as secondary hypothermia caused by severe underlying conditions as revealed by the hypoglycemia status in a cat affected by severe liver dysfunction and heart failure (shock patient). An important role was also played by the cold weather, which negatively influenced body thermoregulation, thus favoring consistent thermal dispersion in a highly weakened cat. Ante-mortem diagnosis of FIP can be difficult. The gold standard for diagnosing FIP is labeling the FCoV antigen in macrophages using IHC in tissues with granulomatous lesions typical of FIP. The clinical presentation described in the present case report was compatible with what has been reported in the literature. 

Signalment, clinical history, BMP, ultrasound investigations and cytological findings were all consistent with a diagnosis of FIP, also confirmed by the molecular genetic test (real-time PCR for feline coronavirus RNA) on the effusive abdominal fluid. The molecular genetic investigation found the FCoV *S* gene single-nucleotide mutation biotype M1058L.

The mild gastrointestinal signs recorded in the cat during the clinical examination at the cat shelter and the severity of the clinical signs observed at the VTH indicate a rapid clinical worsening of the FCoV/FIP infection in a cat also affected by heart failure. 

Cardiac troponin quantification and echocardiography were not performed as other analyses had to be prioritized, as well as due to the Parma municipal multi-cat shelter’s limited budget.

Regarding the heart involvement, impairment of myocardiocyte membrane integrity can result in the release into the extracellular space and blood, within a few hours, of intracellular components, among which are biologically active cytosolic enzymes such as CK and LDH. 

Necropsy revealed pyogranulomatous infiltration of multiple organs including the myocardium, and IHC performed on the myocardial tissue confirmed the presence of FCoV-positive macrophages. FCoV, in the FIP effusive form, could be associated with myocarditis in cats [13]. Prof. A. Corradi, as a co-author both in the present case report and in the article published in 2019 [13], reports that the corpse of the cat investigated in 2018 had been delivered to the Pathology Unit of the Department of Veterinary Science of the University of Parma, for necropsy, in January 2018 (personal data).

FCoV mutation can provide the virus with a different replicative potential and tropism from that observed in typical host cells (i.e., macrophages), so that cardiomyocytes can also become permissive cells [37]. This mutation has already been suggested for SARS-CoV-2 variants that harbor peptides identical to the human cardiac protein [38]. Based on this feature, FCoV with the M1058L mutation can also be considered as a virus biotype that is potentially able to replicate in and is pathogenic for cardiomyocytes. In Sangl’s work, FCoV M1058L variant myocardiotropism was detected in paraffin-embedded heart tissue using genotype-discriminating RT-qPCR, without showing the presence of myocardiocyte lesions [19].

Currently, data on FCoV and SARS-CoV-2 indicate that these viruses have some common epidemiological characteristics (i.e., rapid spread of infection within the population) but show different biological behaviors, such as different target cells for replication and a different p2athogenetic model [39].

## 4. Conclusions

In Sangl’s study [19], the authors reported the presence of the FCoV/FIP M1058L variant in the myocardium in the genetic fingerprinting, without any description of inflammatory/necrotic lesions involving myocardiocytes.

Viral mutations, observed in FCoV and SARS-CoV-2, could represent a new research field worth investigating, to gain insight whether viral mutation also confers a tropism for myocardial cells. Further studies are necessary to support the mutated FCoV M1058L biotype as an uncommon, but possible, causative pathogen of myocarditis in FCoV/FIP-positive cats.

A parallel study, based on statistical analysis, on FCoV/FIP M1058L-positive cases in correlation with heart gross pathology, histopathology, and immunolocalization of the FCoV/FIP M1058L biotype in the myocardium is necessary to determine the real myocardiocyte tropism of the FCoV/FIP M1058L variant. In view of what Sangl and co-workers observed [19], it is imperative to understand whether myocardial lesions are due to inherent virulence of the FCoV/FIP M1058L biotype or whether concomitant causes are required. The potential concomitant causes that could play a role in the occurrence of cardiomyocyte lesions should be investigated with regards to FIP-related or unrelated patient conditions, and it should be studied whether the aforementioned concomitant events can be considered as correlated. The concomitant causes can be related to poor patient conditions or to external distress environments such as cold weather in the winter season. 

However, this is the second report of FCoV/FIP-induced myocarditis in cats and the first report in terms of accurate clinical–pathological case description of myocarditis in a cat positive for the FCoV/FIP M1058L biotype.

## Figures and Tables

**Table 1:** Complete blood count (CBC) at admission.

Parameter	Value	Reference Interval (RI)
RBCs	7.43	5–10 × 10^6^/μL
Hemoglobin	9	8–14 g/dL
Hematocrit	26.3	24–45%
MCV	35.4	41.3–52.6
MCHC	34.2	30–36
Leucocytes	28,300	5000–19,000/mm^3^
Neutrophils	24,340	2000–125,000/μL
Lymphocytes	2480	1500–7000/μL
Monocytes	1200	100–850/μL
Eosinophils	60	0–750/μL
Basophils	20	0/μL
Platelets	20	156–624 × 10^3^/μL

RBCs: red blood cells; MCV: mean corpuscular volume; MCHC: mean corpuscular hemoglobin concentration.

**Table 2:** Serum chemistry at admission.

Parameter	Value	Reference Interval (RI)
CK	5437	91–326 U/L
LDH	1973	63–193 U/L
AST	1226	14–41 U/L
ALT	335	5–45 U/L
SAP	31	19–70 U/L
GGT	1	0–8 U/L
Bilirubin	3.49	0–0.7 mg/dL
Cholesterol	144	64–229 mg/dL
Triglycerides	154	19–81 mg/dL
Creatinine	0.54	0.8–1.80 mg/dL
Urea	99	15–60 mg/dL
Glucose	41	75–160 mg/dL
Total proteins	7.66	6.0–8.0 g/dL
Albumin	1.88	2.10–3.30 g/dL
Phosphorus	7.92	2.9–8.3 mg/dL
Calcium	5.6	7.3–10.5 mg/dL
Sodium	129	141–156 mEq/L
Potassium	3.9	3.6–4.5 mEq/L
Chlorine	112	112–119 mEq/L
Magnesium	3	1.8–2.5 mg/dL
Iron	95	50–118 μg/dL

CK: creatine kinase; LDH: lactate dehydrogenase; AST: aspartate aminotransferase; ALT: alanine aminotransferase; SAP: serum amyloid P component; GGT: gamma-glutamyl transferase.

**Table 3:** Chemical analysis on abdominal effusion at admission.

Parameter	Value
TNCC	6420 cells/mm^3^
Bilirubin	3.02 mg/dL
Creatinine	0.75 mg/mL
Triglycerides	59 mg/dL
Cholesterol	96 mg/dL
Potassium	3.80 mEq/L
Total proteins	5.7 g/dL
Albumin	1.4 g/dL
A/G	0.3

TNCC: total nucleated cell count; A/G: albumin/globulin ratio.

**Table 4:** FCoV molecular genetic testing (PCR) and spike protein mutation (IDEXX laboratories).

Test	Result
Feline coronavirus RNA (real-time PCR)	Positive
Mutation M1058L single-nucleotide variations (SNVs) *S*-RT-rtPCR	Positive
Mutation S1060A single-nucleotide variations (SNVs) *S*-RT-rtPCR	Negative
Biotype	FIPV

**Table 5:** Immunohistochemistry details.

Antibody	Supplier and Code	Clonality	Retrieval	Dilution	Incubation	Labeling System	Visualization
FIPV (feline coronavirus)	BioRad, clone FIPV3-70 (MCA2194)	mouse monoclonal	Buffer H, pH 9.0, in 100 °C water bath	1:2000	1 h 30 min at RT	ABC-peroxidase	DAB

FIPV: feline infectious peritonitis virus; RT: room temperature; ABC: avidin–biotin complex; DAB: 3.3′-diaminobenzidine.

## Data Availability

Data are contained within the article.
